# Effect on enamel shear bond strength of adding microsilver and nanosilver particles to the primer of an orthodontic adhesive

**DOI:** 10.1186/s12903-015-0024-8

**Published:** 2015-03-25

**Authors:** Sonja Blöcher, Roland Frankenberger, Andreas Hellak, Michael Schauseil, Matthias J Roggendorf, Heike Maria Korbmacher-Steiner

**Affiliations:** 1 Department of Orthodontics, University Hospital Giessen and Marburg, Campus Marburg, Georg-Voigt-Strasse 3, Marburg, 35039 Germany; 2 Department of Operative Dentistry and Endodontology, University Hospital Giessen and Marburg, Campus Marburg, Georg-Voigt-Strasse 3, Marburg, 35039 Germany

**Keywords:** Shear bond strength, Microsilver, Nanosilver, Antimicrobial

## Abstract

**Background:**

The objective of this study was to determine whether the addition of microsilver or nanosilver particles to an orthodontic primer affects shear bond strength (SBS) and bracket/adhesive failure.

**Methods:**

Bovine incisors were randomly divided into six groups with 16 specimens in each: In group 1 (control), brackets were bonded with Transbond™ XT primer. In the experimental groups, microsilver (groups 2 and 3) and nanosilver (groups 4–6) particles of different sizes were added to Transbond XT primer and light cured for 15 seconds [group 2: 0.1% (w/w) microsilver particle size 3.5–18 μm; group 3: 0.3% (w/w) microsilver particle size 3.5–18 μm; group 4: 0.11% (w/w) nanosilver particle size 12.6–18.5 nm; group 5: 0.18% (w/w) nanosilver particle size 12.6–18.5 nm; group 6: 0.33% (w/w) nanosilver particle size 12.6–18.5 nm]. Thereafter, brackets were bonded by light curing the adhesive for 20 seconds. After 24 hours of storage in distilled water at 37°C, SBS was measured with a Zwicki 1120 testing machine. The adhesive remnant index and the prevalence of silver spots on the specimen surface were determined under 10× magnification. Statistical two-way analysis of variance was performed to compare SBS, and a chi-square test was used to compare ARI scores and the prevalence of silver spots.

**Results:**

No significant differences in SBS (control: 16.59 ± 6.82 MPa; group 2: 20.6 ± 4.19 MPa; group 3: 16.98 ± 4.84 MPa; group 4: 17.15 ± 5.92 MPa; group 5: 20.09 ± 3.35 MPa; group 6: 16.44 ± 4.51 MPa; *p* > 0.665) and ARI scores (p = 0.901) were found between the control group and any experimental group. Only experimental groups with nanosilver particles revealed statistically more silver spots on the remaining adhesive.

**Conclusions:**

Addition of small concentrations of microsilver or nanosilver particles affects neither SBS nor ARI scores. Addition of nanosilver particles results in silver spots in the remaining primer visible under 10× magnification. Further studies are needed to investigate the anti-caries potential and clinical performance of conventional orthodontic primer with incorporated nanosilver or microsilver particles.

## Background

Demineralization followed by white spot formation is a well-known complication in orthodontic therapy when fixed appliances are used [1-5]. The risk of white spot lesions is significantly less in lingual orthodontics [6], but is still present [7]. It is caused by increased numbers *of Streptococcus mutans* and other pathological microbes in the biofilm, decreased pH and compromised oral hygiene [3]. Preventive measures attempting to reduce demineralization should be independent of the patient’s compliance. These measures include antimicrobial bonding agents, mouth rinses carrying antimicrobial agents, coatings on brackets/wires or remineralizing agents adjacent to orthodontic appliances [5,7].

Lim et al. [8] noted that more bacteria were detected on the adhesive than on the bracket material itself. This fact encouraged the development of innovative antibacterial adhesives designed to reduce bacterial colonization. Recent developments in adhesives have included the incorporation of bioactive glasses into self-mixed resin [9,10] or sealants [11] or other special additives in the adhesive [5,12-15]. Unfortunately, the antimicrobial effect of these additions may persist for only a few weeks [12,16] and may result in higher adhesive failure rates [14,16-18]. Other investigations have dealt with titanium oxide on bracket surfaces [19] or the application of nanoparticles such as titanium, titanium oxide, zinc, zinc oxide, gold, silver or silver ions [5,20-22]. These and other experimental methods have been recently reviewed by Borzabadi-Farahani et al. [17].

However, all new bonding approaches need to fulfill the requirement of acceptable bond strength, which ranges between 5.9 and 7.8 MPa [23].

Silver has long been known as an antimicrobial agent [24] with antimicrobial effects superior to those of gold or zinc [25]. Silver nanoparticles are smaller than 100 nm in size and interact more closely with microbes. They provide a larger surface area for antimicrobial activity owing to a greater surface-to-volume ratio in comparison to larger particles [19,26]. In orthodontics two mechanisms are applied for bacterial reduction: a) combining dental materials with nanoparticles; and b) coating surfaces with nanoparticles to prevent microbial adhesion [17].

Unfortunately, the clinical performance of silver-loaded materials for potentially arresting caries in restorative dentistry has always been limited by discoloration and reduced esthetics [27,28]. Restorative treatment of caries in children with Ketac-Silver, a glass ionomer cement containing 45–55% silver particles [29], resulted in 8.4% with deep marginal discoloration within a 3-year period [27]. Hosoya et al. [28] reported that treatment with silver diamine fluoride resulted in black discoloration of caries-infected primary enamel and dentine.

Therefore, the goal of this *in vitro* study was to evaluate characteristics such as shear bond strength (SBS), bracket/adhesive failure and esthetic performance of Transbond XT™ primer after the incorporation of different sizes of microsilver or nanosilver particles.

## Methods

### Materials

Bovine mandibular incisors were purchased from Rocholl GmbH (Aglasterhausen, Germany), and were checked for cracks and/or caries. Palavit G® was purchased from Heraeus Kulzer GmbH (Wehrheim, Germany), chloramine-T from Sigma Aldrich Chemistry GmbH (Taufkirchen, Germany) and aqua from B. Braun Melsungen AG (Melsungen, Germany). Microsilver™ BG-Med (particle size 3.5–18 μm) was purchased from Bio Gate AG (Nürnberg, Germany), nanosilver AgPure™ W50 (particle size 12.6–18.5 nm) was donated by ras materials GmbH (Regensburg, Germany), discovery® brackets # 790-152-00 for tooth 35 were donated by Dentaurum GmbH (Ispringen, Germany), Transbond™ XT primer and adhesive were purchased from 3 M Unitek Orthodontic Products (Monrovia, CA, USA), Ormco® etching gel was purchased from Ormco (Orange, CA, USA) and Zircate® Prophy Paste was purchased from Dentsply DeTrey GmbH (Konstanz, Germany). All chemicals were stored according to the manufacturer’s instructions.

All materials are listed in Table 1.

**Table 1:** **Materials used in this study**

Material	Manufacturer
Bovine mandibular incisors	Rocholl GmbH; Aglasterhausen; Germany
Palavit G®	Heraeus Kulzer GmbH; Werheim; Germany
Microsilver™ BG-Med (particle size 3.5–18 μm)	Bio Gate AG; Nuremberg; Germany
Nanosilver AgPure™ W50 (particle size 12.6–18.5 nm)	ras materials GmbH; Regensburg; Germany
Discovery® brackets # 790-152-00 for tooth 35	Dentaurum GmbH & Co. KG; Ispringen; Germany
Transbond™ XT primer and adhesive	3 M Unitek Orthodontic Products; Monrovia; CA; USA
Ormco® etching gel	Ormco Corporation; Orange; CA; USA
Zircate® Prophy Paste	Dentsply DeTrey GmbH; Konstanz; Germany
Chloramine-T hydrate	Sigma Aldrich Chemistry GmbH; Taufkirchen/Munich; Germany
Aqua	B. Braun Melsungen AG; Melsungen; Germany

### Specimens and preparation of stock solutions of microsilver and nanosilver

Bovine mandibular incisors were embedded in Palavit G® chemically cured resin. The labial surface was positioned facing up and parallel to the resin. The teeth were stored in 0.5% chloramine-T solution.

For medical application of microsilver particles a range of 0.1–0.5% w/w is recommended by the manufacturer [30]. According to the manufacturer’s recommendations, 500 ppm (0.05% particles absolute or 0.11% weight/weight [w/w]) nanosilver particles should be used for significant bacterial inhibition for coatings, 800 ppm (0.08% particles absolute or 0.18% w/w) on medical devices and 1500 ppm (0.15% particles absolute or 0.33% w/w) for strong bacterial reduction on medical devices [31]. Therefore, the addition of nanosilver particles to Transbond™ XT primer in this study is based on these recommendations.

To compare the *in vitro* performance of microsilver and nanosilver particles, the same w/w concentrations were chosen within the recommended concentration range.

Stock solutions of 10% (w/w) microsilver and 11% (w/w) nanosilver in aqua were prepared. Prior to the bonding procedure, the stock solutions were diluted in Transbond™ XT primer and mixed for preparing the working solutions. The solutions were diluted as follows:

- 0.1% (w/w) microsilver solution:
  - 1:100 dilution (1 μl microsilver 10% [w/w] stock solution and 99 μl primer)
- 0.3% (w/w) microsilver solution:
  - 3:100 dilution (3 μl microsilver 10% [w/w] stock solution and 97 μl primer)
- 0.11% (w/w) nanosilver solution:
  - 1:100 dilution (1 μl nanosilver 11% [w/w] stock solution and 99 μl primer)
- 0.18% (w/w) nanosilver solution:
  - 1.8:100 dilution (1.8 μl nanosilver 11% [w/w] stock solution and 98.2 μl primer)
- 0.33% (w/w) nanosilver solution:
  - 3:100 dilution (3 μl nanosilver 11% [w/w] stock solution and 97 μl primer)

For SBS testing the teeth were randomly divided into six groups of 16 samples each:

- Group 1: primer, control group

Experimental groups 2–6

- Group 2: primer with 0.1% (w/w) microsilver (particle size 3.5–18 μm)
- Group 3: primer with 0.3% (w/w) microsilver (particle size 3.5–18 μm)
- Group 4: primer with 0.11% (w/w) nanosilver (particle size 12.6–18.5 nm)
- Group 5: primer with 0.18% (w/w) nanosilver (particle size 12.6–18.5 nm)
- Group 6: primer with 0.33% (w/w) nanosilver (particle size 12.6–18.5 nm)

### Bonding procedure

Group 1 (primer, control group): Teeth were polished with Zircate® Prophy Paste, rinsed with water and air-dried. The enamel surfaces were then etched for 30 seconds with a 37% phosphoric acid etching gel, then rinsed for 10 seconds with water and air-dried. A thin film of primer was applied on the etched enamel surface, and illuminated with a light source (Poly Lux II, KaVo Dental, Biberach/Riss, Germany) for 15 seconds. Then, Transbond XT adhesive was applied to the bracket base, the bracket was applied and pressed onto the enamel surface, and excessive adhesive was removed prior to polymerization, which was conducted for 20 seconds each from the mesial and distal sides.

Groups 2 to 6 (primer with microsilver or nanosilver, experimental groups): The procedure was the same as in group 1, but instead of pure primer, 0.1% (w/w) or 0.3% (w/w) microsilver primer mixture or 0.11% (w/w), 0.18% (w/w) or 0.33% (w/w) of nanosilver primer mixture were used. The primer mixture was thoroughly mixed with a brush and then applied to each tooth.

The bonding procedure was performed by one investigator (SB) according to the manufacturer’s instructions. All teeth were bonded with discovery® lower premolar brackets with a laser-structured base; these brackets are often used as reference according to the DIN standard 13990 [32]. The average surface area of the bonded bracket was 13.42 mm^2^.

**Figure 1:**
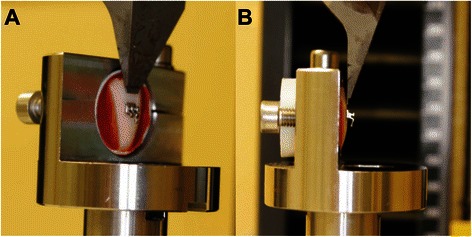
**Representative photographs of a tooth positioned in the testing machine. A**: lateral side view; **B** lateral view.

A representative photograph (Canon EOS650D camera) of a bonded tooth in the testing machine is shown in Figure 1.

### Shear bond strength measurement

Shear bonding strength was measured after 24 hours of storage in distilled water at 37°C with a Zwicki 1120 testing machine (Zwick Roell, Ulm, Germany). A force was applied to the bracket base at the wings in an occlusogingival direction with a crosshead speed of 1 mm/min. The force was measured in Newtons (N). SBS values were calculated by converting Newtons into megapascals (MPa).

### Assessment of residual adhesive

The amount of residual adhesive adhering to the enamel surface was quantified by using the adhesive remnant index (ARI) developed by Årtun and Bergland [33]. The ARI scores of all samples were recorded twice by the same investigator using an optical stereomicroscope (Leica Z 6 APO, Leica Microsystems, Wetzlar, Germany) under 10× magnification. Scoring groups are: 0, no adhesive remains on the tooth; 1, less than 50% of the adhesive remains on the tooth; 2, more than 50% of the adhesive remains on the tooth; 3, all adhesive remains on the tooth.

For scanning electron microscope (SEM) analysis of the adhesive remnants the samples were sputtered with gold/platinum in an Edwards sputter coater S150 B (Munich, Germany) and analyzed by SEM image (Phenom FEI G1 and Phenom Software Prosuite, Eindhoven, The Netherlands).

### Assessment of silver spots after debonding

After debonding, the tooth surfaces were inspected by eye and under 10× magnification with an optical stereomicroscope for discoloration [28,29].

### Statistical analysis

Statistical analysis was performed using SPSS 21.0.0 software (SPSS Inc., Chicago, IL).

For sample size calculation for the SBS measurements, a power analysis for ß-error (power > 80) was performed.

The SBS data were analyzed using the Kolmogorov–Smirnov test, followed by analysis of variance (ANOVA) and a Kaplan–Meier survival analysis.

The ARI data were analyzed using the Kolmogorov–Smirnov test, followed by the chi-square test. Silver spot analysis was performed using the Kolmogorov–Smirnov test, followed by the chi-square test. Additionally chi-square tests were used to analyze inter-group differences. *P* values less than 0.05 were considered statistically significant.

## Results

### Sample size calculation

The power for these 16 samples was 0.818. For this power a minimum of 15 samples per group was needed.

### Shear bond strength measurement

SBS values were as follows: group 1 (control): 16.59 ± 6.82 MPa; group 2 (primer + 0.1% microsilver): 20.6 ± 4.19 MPa; group 3 (primer + 0.3% microsilver): 16.98 ± 4.84 MPa; group 4 (primer + 0.11% nanosilver): 17.15 ± 5.92 MPa; group 5 (primer + 0.18% nanosilver): 20.09 ± 3.35 MPa; group 6 (primer + 0.33% nanosilver): 16.44 ± 4.51 MPa.

**Figure 2:**
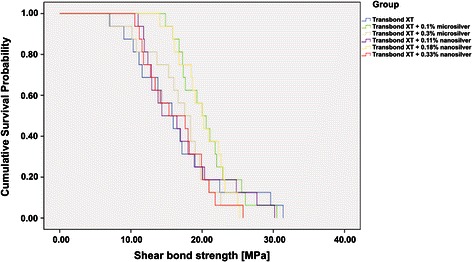
**Kaplan–Meier survival analysis for all experimental groups.**

The Kolmogorov–Smirnov test for SBS showed normal distribution in all experimental groups (statistic = 0.045, df = 96, mean square = 2.941, F = 0.905, *p* = 0.200). Therefore, ANOVA was applied. Two-way ANOVA showed no statistically significant difference for SBS between the experimental groups and the control group (*p* > 0.665). In general, no significant differences could be detected between the groups as well as by 2-by-2-comparisons of all groups. Figure 2 shows the results of the Kaplan–Maier survival analysis. Descriptive statistics and the results of the ANOVA test are presented in Table 2.

**Table 2:** **Descriptive statistics of the groups and comparison of SBS values (ANOVA)**

Groups	n	Mean (sd) [MPa]	Range [MPa]	95% CI [MPa]	Median [MPa]	95% CI [MPa]
TB	16	16.59 (6.82)	7.03–31.38	13.25–19.94	15.76	12.55–18.97
TB + 0.1% (w/w) μAg	16	20.6 (4.19)	14.86–30.49	18.55–22.66	20.00	17.45–22.55
TB + 0.3% (w/w) μAg	16	16.98 (4.84)	6.9–25.78	14.61–19.35	17.55	14.14–20.96
TB + 0.11% (w/w) nAg	16	17.15 (5.92)	11.02–30.18	14.25–20.05	14.34	9.15–19.53
TB + 0.18% (w/w) nAg	16	20.09 (3.35)	14.05–25.28	18.45–21.74	19.99	17.66–22.32
TB + 0.33% (w/w) nAg	16	16.44 (4.51)	10.55–25.74	14.23–18.65	15.34	8.48–22.20

ANOVA: Sum of squares = 273.5, df = 93, mean square = 2.941, *F*-value = 0.905, *P*-value = 0.665.

### Assessment of residual adhesive

Table 3 presents the ARI scores. The 2-fold determination of ARI scores on two different days showed no differences at all, and the applied Dahlberg formula generated an error of zero [34].

**Table 3:** **Adhesive remnant index (ARI)**

	ARI score					Group differences
Groups	0	1	2	3	Median	
TB	0	7	6	3	2	A
TB + 0.1% (w/w) μAg	0	9	4	3	1	A
TB + 0.3% (w/w) μAg	0	8	6	2	1	A
TB + 0.11% (w/w) nAg	0	6	6	4	2	A
TB + 0.18% (w/w) nAg	0	7	4	5	2	A
TB + 0.33% (w/w) nAg	0	7	5	4	2	A

ARI scores 0, no adhesive remains on tooth; 1, less than 50% of adhesive remains on tooth; 2, more than 50% of adhesive remains on tooth; 3, all adhesive remains on tooth.

All groups that are not significantly different from each other are shown with the same letters (chi-square test = 1.599, df = 5, *p* = 0.901).

There were no instances of an ARI score of 0 representing no adhesive on the tooth. The median ARI score for the control and all experimental groups with nanosilver particles was 2, while the experimental groups with microsilver particles revealed a median of 1. The Kolmogorov–Smirnov test showed normal distribution for the ARI scores (Statistic = 0.290, df = 96, *p* = 0.772). The chi-square test between all tested groups showed no statistical difference (p = 0.901).

### Assessment of discoloration after debonding

**Figure 3:**
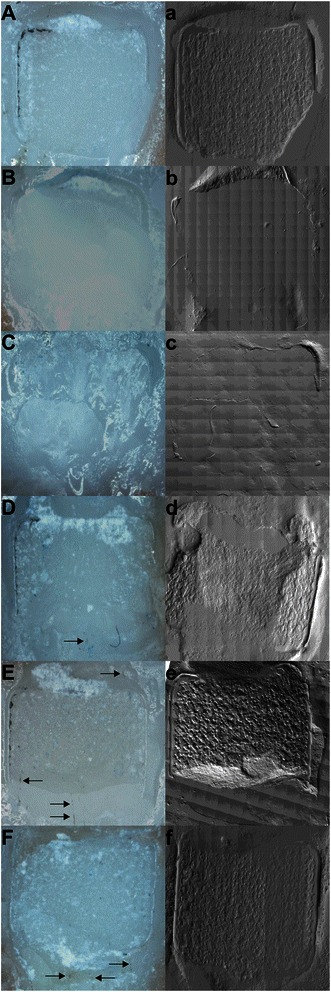
**Representative microscopic and SEM images of median ARI scores. A**-**F**: 10× magnification; arrows mark detected silver spots. **a**-**f** SEM counterparts (45×) of the same sample. **A** and **a**, primer (Transbond XT); **B** and **b**, primer and 0.1% (w/w) microsilver; **C** and **c** primer and 0.3% (w/w) microsilver; **D** and **d**, primer and 0.11% (w/w) nanosilver; **E** and **e**, primer and 0.18% (w/w) nanosilver; **F** and **f**, primer and 0.33% (w/w) nanosilver.

By visual eye check no silver spots were visible on any tooth. Under 10× stereomicroscopic magnification small silver spots were detected on the resting primer/adhesive on the tooth surface in all experimental groups (Table 4, Figure 3).

**Table 4:** **Number of specimens revealing silver spots detected under 10× magnification**

Groups	Number of specimens	Group difference
TB	0	A
TB + 0.1% (w/w) μAg	2	A
TB + 0.3% (w/w) μAg	1	A
TB + 0.11% (w/w) nAg	4	B
TB + 0.18% (w/w) nAg	6	B
TB + 0.33% (w/w) nAg	9	B

Groups with the same letters are not statistical different (chi-square test letter A: chi-square = 2.089, df = 2, *p* = 0.352; chi-square test letter B: chi-square = 3.241, df = 2, *p* = 0.198). Chi-square test on all groups shows statistically significant difference (chi-square = 20.074, df = 5, *p* = 0.01).

The Kolmogorov–Smirnov test showed no normal distribution for silver spots (Statistic = 0.142, df = 96, *p* = 0.000). The chi-square test between all tested groups showed a statistically significant difference (chi-square = 20.074, df = 5, *p* = 0.01), revealing significantly more teeth with silver spots in the experimental groups with applied nanosilver particles when compared with the control or the experimental groups with incorporated microsilver particles. The chi-square test between primer, primer and 0.1% (w/w) microsilver and primer and 0.3% (w/w) microsilver showed no statistically significant difference (*p* = 0.352). The chi-square test between primer and 0.11% (w/w) nanosilver, primer and 0.18% (w/w) nanosilver and primer and 0.33% (w/w) nanosilver was statistically significantly different to the control and the experimental groups with added microsilver particles (*p* = 0.001).

## Discussion

Based on our *in vitro* results, neither SBS nor ARI scores were significantly affected by the addition of microsilver or nanosilver particles of different sizes. Ahn et al. [20] added 250 ppm and 500 ppm of silver nanoparticles with a size smaller than 5 nm in combination with nanosized silica particles to self-mixed experimental composite adhesives. They found that SBS values measured on human premolars did not significantly differ between the experimental composite adhesives and conventional adhesives. Although we added silver nanoparticles more extensively and at greater concentrations than Ahn et al. [20], we found that our SBS results were comparable with theirs [20]. Following our experimental procedure, Akhavan et al. [21] added silver nanoparticles to Transbond™ XT primer; however, they used higher concentrations (1%, 5% and 10%) of silver nanoparticles and added 5% hydroxyapatite to the mixtures. Furthermore, they measured SBS on human premolars with a crosshead speed of 0.5 mm/min making it impossible to compare their SBS values with ours [35]. Sadat-Shojai et al. [36] described the influence on bond strength of nanoparticles incorporated into dentin bonding materials. SBS increased with the incorporation of 0.2% hydroxyapatite nanoparticles and later decreased at higher concentrations [36]. The authors discussed whether the higher concentrations of nanoparticles would aggregate and therefore interact with the nanomaterial, which could again lead to defects in the matrix. According to Sadat-Shojai et al. [36] and Ahn et al. [20], concentrations of nanosilver particles of up to 0.33% (w/w) do not interfere with the matrix of the primer or the adhesive. The concentration used by Akhavan et al. [21] could be in a range that could possibly affect the matrix.

The recorded ARI scores varied between 1 and 3 in the different groups and did not significantly differ between the control and experimental groups. Neither the incorporation of microsilver particles nor nanosilver particles affected the bracket/adhesive failure. O’Brien et al. [37] found that the evaluation of ARI score is quite subjective. Therefore, ARI scores were measured twice. We found no differences in these two determinations.

Many studies have investigated nanosilver particles and their potential effects on bacteria or animal cells [24,38-43]. In our *in vitro* study we did not investigate the release of nanosilver into saliva, but these studies should be considered. The examined microparticulate silver is not cytotoxic and is certified for medical applications (ISO 10993–5) [44,45]. Bürgers et al. [46] in an *in vitro study* applied microsilver to the resin composite X-Flow (Dentsply De Trey). They found significant anti-adherent and antimicrobial effects on the composite surface [46].

Silver or silver compounds have been repeatedly added to restorative materials in restorative dentistry [27,28,47]. Unfortunately, the use of these materials resulted in discolored restorations and/or teeth [28,29,47]. Kawasaki et al. [47] compared the protective effect of diamine silver fluoride with ammonium hexafluorosilicate on the demineralization of dentine. Diamine silver fluoride produced a shallower demineralized depth, but it stained the teeth black owing to sulfonization. Investigating the location of the two solutions, they found that the silver of the diamine silver fluoride covered the surface of the mineral and the silicium of the ammonium hexafluorosilicate was located in the mineral lesion [47]. In our study, visible silver discoloration was not detected after debonding. However, by using 10× magnification, we found teeth with lightly scattered silver spots in the area of residual primer/adhesive. Adding silver microparticles (particle size 3.5–18 μm) to the primer resulted in a slight appearance of silver spots without any statistically relevant impact. The number of teeth with silver spots increased significantly with the addition of silver nanoparticles (particle size 12.6–18.5 nm). Cheng et al. [48] added quaternary ammonium and silver nanoparticles to the primer of Scotchbond Multi-Purpose adhesive. They noted that this primer had esthetics/color similar to those of the control [48]. These authors concluded that the high surface area of the silver nanoparticles provided a potent effect at a low filler level to avoid negative influence on color and mechanical properties. In a second study, Cheng et al. [49] added silver nanoparticles (particle size 2.7 nm) to amorphous calcium phosphate-containing resin. They found that the addition of 0.042% silver nanoparticles imparted no influence on color or flexural strength. Higher concentrations of 0.175% revealed a brownish color and a drop in strength. Therefore, they recommended the addition of only a low concentration of silver nanoparticles. Besinis et al. [50] recently applied a silver nanoparticle solution (particle size 56.8 ± 18.6 nm) and a silver nitrate solution (particle size 52.8 ± 18.6 nm) to human dentine discs. Both solutions exhibited an antibacterial effect [50], but only the silver nanoparticle solution achieved a clinically acceptable color match, while the silver nitrate solution produced esthetically unacceptable results [50]. Further studies are needed to investigate whether spots observed in this *in vitro* study can be removed by cleaning after debonding. If so, the incorporation of silver particles could be an opportunity to reduce bacterial colonization during orthodontic therapy.

We used bovine incisors for this *in vitro* study, owing to the difficulty of obtaining intact human teeth of sufficient quality and quantity. This usage is acceptable for bonding studies instead of human teeth according to DIN 13990 [32], and these teeth are often used in studies [51]. There are similarities between bovine and human enamel in crystallite orientation, the dimensions of the outer prisms, and the enamel matrix protein composition [52]. However, there are also differences: bovine enamel contains some different chemical elements [53], and has a different prism arrangement, thicker crystallites, a wider interprismatic region, and increased porosity [53-57]. Although Reeh et al. [52] found a similar lubricity between human and bovine enamel, these differences resulted in greater microleakage of bovine enamel [53].

In accordance with other studies Transbond™ XT was chosen as the control adhesive [9,12,22] because it is viewed as the orthodontic gold standard adhesive [21].

We used discovery® brackets because they are often used as reference brackets according to the DIN standard 13990 [32]. Therefore, the results of our *in vitro* study are limited to our study design that was widely based on the DIN standard 13990.

Further studies are needed to investigate a) if these *in vitro* results can be confirmed under *in vivo* conditions and b) if the nanosilver and/or microsilver particles incorporated in the Transbond™ XT primer show anti-caries activity under clinical conditions, and c) if the clinical performance in terms of discoloration is acceptable.

## Conclusions

The addition of small amounts of antibacterial silver microparticles or nanoparticles affects neither the SBS nor the bracket/adhesive failure of Transbond™ XT primer. Further *in vivo* studies on human teeth are needed to examine if the incorporation of microsilver or nanosilver particles in orthodontic primers can reduce bacterial colonization and white spot formation without discoloration of the teeth.

## Abbreviations

ANOVA: Analysis of variance
ARI: Adhesive remnant index
μl: Microliter (10^−6^ liter)
μm: Micrometer (10^−6^ meter)
mm: Millimeter (10^−3^ meter)
mm^2^: Square mm
MPa: Megapascal
N: Newton
nm: Nanometer (10^−9^ meter)
ppm: Parts per million
SBS: Shear bond strength
SEM: Scanning electron microscope
w/w: Weight/weight
